# An index-based algorithm for fast on-line query processing of latent semantic analysis

**DOI:** 10.1371/journal.pone.0177523

**Published:** 2017-05-16

**Authors:** Mingxi Zhang, Pohan Li, Wei Wang

**Affiliations:** 1 College of Communication and Art Design, University of Shanghai for Science and Technology, Shanghai, China; 2 School of Computer Science, Fudan University, Shanghai, China; Tianjin University, CHINA

## Abstract

Latent Semantic Analysis (LSA) is widely used for finding the documents whose semantic is similar to the query of keywords. Although LSA yield promising similar results, the existing LSA algorithms involve lots of unnecessary operations in similarity computation and candidate check during on-line query processing, which is expensive in terms of time cost and cannot efficiently response the query request especially when the dataset becomes large. In this paper, we study the efficiency problem of on-line query processing for LSA towards efficiently searching the similar documents to a given query. We rewrite the similarity equation of LSA combined with an intermediate value called partial similarity that is stored in a designed index called partial index. For reducing the searching space, we give an approximate form of similarity equation, and then develop an efficient algorithm for building partial index, which skips the partial similarities lower than a given threshold *θ*. Based on partial index, we develop an efficient algorithm called ILSA for supporting fast on-line query processing. The given query is transformed into a pseudo document vector, and the similarities between query and candidate documents are computed by accumulating the partial similarities obtained from the index nodes corresponds to non-zero entries in the pseudo document vector. Compared to the LSA algorithm, ILSA reduces the time cost of on-line query processing by pruning the candidate documents that are not promising and skipping the operations that make little contribution to similarity scores. Extensive experiments through comparison with LSA have been done, which demonstrate the efficiency and effectiveness of our proposed algorithm.

## Introduction

Many real data sets could be grouped as documents, including as web pages, literature and product profiles. With such data sets becoming massive and diverse, there is a need for designing algorithmic tools and developing applications to discover the underlying relationship from the data. Consider an example of the document search in a dataset, even though a document is on precisely the same topic to a input query of keywords, it may not be searched when its contained terms are different to the input keywords. In previous work, there are some semantic approaches that can be used finding the documents whose semantic is similar to the query of keywords, e.g., Latent Semantic Analysis (LSA) [1–4], Probabilistic LSA (PLSA) [5, 6], Latent Dirichlet Allocation (LDA) [7–10] and latent factorization model (LFM) [11, 12]. Among these approaches, LSA is a well-known representative which has been widely applied to various research fields, including document retrieval [13, 14], query expansion [15, 16], data extraction [17, 18] and text classification [19, 20]. For improving the performance of these applications, LSA provides an effective function for searching the similar documents for a given query of keywords. Specifically, LSA represents the relationship between documents and terms by a term-document matrix that is further decomposed into a product of three other matrices by the singular value decomposition (SVD) [1, 3, 4]. SVD is the mathematical tool behind LSA and some applications including association prediction [21], similarity computation [22, 23], clustering [24, 25], images analysis [26] and collaborative filtering [27, 28]. For the given query, LSA transforms it into a pseudo document vector and computes the similarities between query and candidate documents over the SVD result of the term-document matrix.

LSA has also been applied to other research fields recently, including social data analysis [29, 30], collaborative filtering [31–33], sign language translation [34] and gene sequence analysis [35–40]. For example, in the field of social data analysis, [29] adopted LSA for producing better annotated video clip in social multimedia data. [30] measured the semantic similarity in the text of social media by using a topic-based LSA. For obtaining better result of recommendation, [31] proposed a latent class regression recommender system (LCRRS) through extending PLSA for collaborative filtering based on cluster-wise linear regression. [32] presented a component recommender approach based on LSA to initialize the word distributions for different topics. [33] fused LSA and K-means for better recommendation of antiarrhythmic drugs through capturing the latent factors between the arrhythmia types and patients. In the field of sign language translation, [34] used de-bruijn graph with LSA in the decoding process to improve the quality and accuracy of translation result. For discovering the associations between genes and diseases, [35] computed the similarities between genes using LSA algorithm, and divided the similar cardiovascular disease (CVD) association genes into different clusters. [36] applied the latent factorization model (LFM) to predict the genes related to diseases by representing the relationship between genes and diseases with the gene-disease association matrix [37, 38]. [39] employed LSA to visualize gene expression experiments and defined an asymmetric similarity measure of association for genes by using the correspondence word-gene document-experiment. [40] presented a text mining approach based on LSA for prioritization, clustering and functional annotation of miRNAs.

Nevertheless, although LSA yields promising similar results and provides an effective solution to above applications, however, lots of unnecessary operations are involved in similarity computation and candidate check during on-line query processing, which make it expensive in terms of time cost and cannot efficiently response the query request especially when the dataset grows large.

Some optimization techniques on LSA have been developed recently. [41] proposed a faster optimization algorithm for solving the Non-negative Sparse Latent Semantic Analysis (NN-Sparse LSA), and implemented the parallel version of the fast NN-Sparse LSA algorithm by parallel programming framework of the Compute Unified Device Architecture (CUDA). [42] proposed an on-line belief propagation for PLSA to handle big data streams by splitting the data stream into a set of small segments and uses the estimated parameters of previous segments to calculate the gradient descent of the current segment. [43] proposed a randomized SVD algorithm that scales the original matrix to a small matrix by sampling a constant number of rows or columns of the matrix, and the SVD of the original matrix is derived approximately by computing the SVD of the small matrix. [44] proposed an incremental SVD algorithm, which can update the SVD of a given matrix dynamically by adding rows and columns of data without re-computing the SVD result. [45] proposed an algorithm for incrementally computing the left singular vectors of SVD result by exploiting the relationship between the *QR*-decomposition and the SVD. QUIC-SVD [46] provides an algorithm which producing the approximation of the whole-matrix SVD based on a sampling mechanism called the cosine tree, and provides speedups of several orders of magnitude over exact SVD. [47, 48] proposed an algorithm for accurately computation of SVD by inhering the high accuracy properties of the Jacobi algorithm [49]. [50] introduced a bi-iteration type subspace tracker for updating SVD approximation of the cross-correlation matrix of dimension *N* × *M*. [51] designed a secure, correct, and efficient protocols for outsourcing the SVD of a malicious cloud. [52] proposed an algorithm for extremely fast dimensionality reduction by employing the Gaussian-based random projection and a Hadamard-based random projection. However, the above approaches mainly focus on improving the efficiency in the pre-computation stage, few of them pay attention to the efficiency problem of on-line query processing.

In this paper, we study the efficiency problem of on-line query processing for LSA, towards efficiently searching the similar documents in large dataset. We rewrite the similarity equation of LSA combined with an intermediate value called partial similarity, and divide the similarity computation into two steps: the first step is to compute the partial similarities, and the second step is to compute the similarities between query and candidate documents based on the partial similarities. The partial similarities are computed in the off-line stage and stored in a designed index called partial index. For reducing the searching space during query processing, we give an approximate form of similarity equation, and then develop an efficient algorithm for building partial index, which skips the partial similarities lower than a given threshold *θ*. The similarities between query and candidate document is computed in the on-line stage, and an efficient algorithm called ILSA is developed for supporting fast on-line query processing through searching similar documents from the partial index. For a given query of keywords, we first transform it into a pseudo document vector and then compute the similarities between query and candidate documents by accumulating the partial similarities obtained from the partial index. ILSA accesses only the partial index nodes corresponds to non-zero entries in the pseudo document vector, which prunes candidate documents that are not promising and reduces the unnecessary operations on similarity computation that make little contribution to similarity scores. By extensive mathematical analysis, we give the maximal upper bound of the difference between ILSA and naive LSA under threshold *θ*. Extensive experiments through comparison with LSA have been done, which demonstrate the efficiency and effectiveness of our proposed algorithm.

## Methods

### Preliminaries

Before we discuss further on LSA, we first list the definition of correlation matrix of term-document for the subsequent discussions.

**Definition 1** (Correlation Matrix of Term-Document). *A Correlation Matrix of Term-Document is formalized as a* *M* × *N* *matrix* *C*_*M* × *N*_, *where* *M* *is size of term set* *T* *and* *N* *is the size of document set* *D*. *In which, the entry* $C_{t_{i},d_{j}}$ *represents the correlation between term* *t*_*i*_ *and document* *d*_*j*_, *which is initialized as the number of times that term* *t*_*i*_ *occurs in document* *d*_*j*_.

LSA maps each document into a *M*-dimension vector and forms a correlation matrix of term-document *C*. Unlike precise matching method, the matrix *C* is decomposed by SVD, that compresses matrix *C* into a new low-dimension space to remove the noise terms. SVD can not only reduce the scale of the data, but also find the underlying relationship between terms. During the on-line query processing, the input terms are firstly transform into a query vector of pseudo document, and then LSA uses cosine coefficient to compute the similarity between query vector and the low-dimension vector corresponds to each document over the decomposition result of matrix *C*. The candidate documents are sorted according to the corresponding similarities, and then returned to current user. Besides cosine, other measure can also be used for computing similarity, such as Jaccard coefficient and dot product, and without loss of generality we choose cosine to measure the similarity. Specifically, the procedure of LSA can be summarized as follows.

1. Building term-document correlation matrix *C* by analyzing document set *D*. For each document *d*_*i*_ ∈ *D*, transform it into vector form *V*_*i*_(*v*_1_, *v*_2_, …, *v*_*M*_), where *v*_*j*_ refers to $C_{t_{i},d_{j}}$ as described in Definition 1, that is computed by counting the number of times that term *t*_*j*_ occurs in document *d*_*i*_. Precisely, *v*_*j*_ is usually defined by the normalized TF*IDF (term frequency inverse * document frequency) model [53, 54] that is widely used for measuring the term weights in a document set [55, 56]. Specifically, the entry $C_{t_{i},d_{j}}$ is assigned as the TF*IDF of term *t*_*i*_ that occurs in document *d*_*j*_. After normalizing vector *V*_*i*_ for each document *d*_*i*_ ∈ *D*, the term-document correlation matrix *C* is represented as:
  $$C=(V_{1},V_{2},\ldots ,V_{N})$$ (1)
2. Singular value decomposition (SVD) of term-document correlation matrix *C*. For a term-document correlation matrix *C*, there exists a decomposition such that
  $$C=USV^{T}$$ (2)
  where *U* is an *M* × *M* matrix, the column of *U* is the orthogonal vector of matrix *CC*^*T*^, and *C*^*T*^ is the transpose of *C*; *S* is an *M* × *N* matrix, $S_{i,i}=\sqrt{\lambda_{i}}$, and *λ*_*i*_ is the *i*-th biggest eigenvalue of *CC*^*T*^; and *V* is an *N* × *N* matrix, the vector of *V* is the orthogonal vector of matrix *C*^*T*^ *C*, and *V*^*T*^ is the transpose of *V*.
3. Get low rank approximation matrix of matrix *C*. The *r*-dimension rank approximation matrix of *C* can be described as:
  $$C_{r}=U_{r}S_{r}V_{r}^{T}$$ (3)
  where *U*_*r*_ and *V*_*r*_ are calculated by discarding the columns of *U* and *V* from *r* + 1 on, *S*_*r*_ are calculated by discarding both columns and rows from *r* + 1 on, and *r* ≪ *M*. The noisy terms can be removed by setting *r*, but some informative terms would be ignored when *r* is set too small. On the other hand, when *r* is set too big, some noisy terms would be involved.
4. On-line query processing for input keywords. Given a query *Q* of keywords, the procedure of on-line query processing is described as follows. First, view this as a vector of a mini document and transform it into a pseudo document vector $\hat{Q}$ of low-dimensional space according to the result of SVD, described as:
  $$\hat{Q}=S_{r}^{-1}U_{r}^{T}Q$$ (4)
  where $S_{r}^{-1}$ is the inverse matrix of *S*_*r*_. Second, compute the similarity between *Q* and document *d*_*i*_ ∈ *D* by the cosine value between $\hat{Q}$ and the column vector *V*^*T*^(:, *i*), described as:
  $$\text{sim}\left(Q,d_{i}\right)=\text{cosine}\left(\hat{Q},V_{r}^{T}\left(:,d_{i}\right)\right)=\frac{\sum_{t_{j}}\hat{Q}\left(t_{j}\right)V_{r}^{T}\left(t_{j},d_{i}\right)}{\sqrt{\sum_{t_{j}}{\left(\hat{Q}\left(t_{j}\right)\right)}^{2}}\sqrt{\sum_{t_{j}}{\left(V_{r}^{T}\left(t_{j},d_{i}\right)\right)}^{2}}}$$ (5)
  And finally, find top *k* most similar documents ranking from the document set such that sim(*Q*, *d*_*i*_) ≥ sim(*Q*, *d*_*x*_) for *d*_*i*_ in the returning list and *d*_*x*_ not, and then sort them with similarities descending in the returning list.

### Rewrite LSA similarity equation

During on-line query processing of LSA, two factors that increase the computational cost are involved. First, the more candidates to check, the more time the algorithm will take; and second, when computing the similarity between the query and each candidate, the more terms related to the candidate, the more time will take. Therefore, the intuition to speed up the search is to prune the candidates that are not promising and reduce the unnecessary operations that make little contribution to similarity scores.

For optimizing the on-line query processing, we next rewrite the similarity equation of LSA equivalently based on Eq (5), described as:

$$\text{sim}\left(Q,d_{i}\right)=\frac{\sum_{t_{j}}\hat{Q}\left(t_{j}\right)\text{PartialSim}\left(d_{i},t_{j}\right)}{\sqrt{\sum_{t_{j}}{\left(\hat{Q}\left(t_{j}\right)\right)}^{2}}}$$ (6)

where PartialSim(*d*_*i*_, *t*_*j*_) is defined as:

$$\text{PartialSim}\left(d_{i},t_{j}\right)=\frac{V_{r}^{T}\left(t_{j},d_{i}\right)}{\sqrt{\sum_{t_{j}}{\left(V_{r}^{T}\left(t_{j},d_{i}\right)\right)}^{2}}}$$ (7)

which is called the partial similarity between document *d*_*i*_ and term *t*_*j*_. Based on this equation, the LSA similarity computation can be divided into two steps: the first step is to compute the partial similarities between documents and terms, and second step is to compute the similarity scores based on the partial similarities.

### Partial index

**Fig 1:**
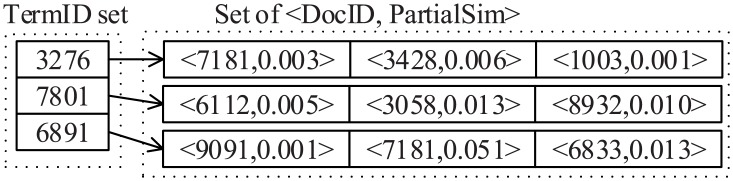
Example of partial index.

We next introduce an index, called partial index, for reducing the searching space of LSA. The partial index used for storing the partial similarity scores in order to reduce the candidate size and optimize similarity computation. The spiritual of the partial index is similar to the pruning index proposed in our previous work in [57, 58]. An example of partial index is shown as Fig 1, where TermID denotes the term ID, DocID denotes document ID, PartialSim denotes the partial similarity, and the two-tuple 〈DocID,PartialSim〉 describes that the partial similarity between a document DocID and a term TermID that the document DocID belongs to is PartialSim. For example, in the set of “3276”, the 〈7181, 0.003〉 describes that the partial similarity between document “7181” and term “3276” is 0.003, and in the set of “7801”, the 〈3058, 0.013〉 describes that the partial similarity between document “3058” and term “7801” is 0.013.

Formally, the partial index is represented by a set $I=\cup_{i=1}^{\left|T\right|}\left\{I\left(t_{j}\right)\right\}$, where *I*(*t*_*j*_) = {〈*d*_*i*_, PartialSim(*d*_*i*_, *t*_*j*_)〉|*d*_*i*_ ∈ *D*∧〈*d*_*i*_, PartialSim(*d*_*i*_, *t*_*j*_)〉 ≠ 0}. In which, 〈*d*_*i*_, PartialSim(*d*_*i*_, *t*_*j*_)〉 is a node of the partial index corresponds to the 2-tuple of 〈DocID, PartialSim〉 form. Specifically, *d*_*i*_ is the document corresponds to DocID and PartialSim(*d*_*i*_, *t*_*j*_) is the partial similarity between document *d*_*i*_ and term *t*_*j*_ corresponds to PartialSim.

### Approximate form of partial index

In fact, not all the terms are informative to represent the documents. For example, “SimRank: A Measure of Structural-Context Similarity” is a paper on the topic of structural-based similarity measure, so it is usually high relevant to the terms “SimRank”,“link”, “LinkClus”, “similarity” and etc., and low or not relevant to terms “phisical”, “astronomy” and etc. During on-line query processing, the lower or not relevant terms would decrease the on-line query processing efficiency and even affect the quality of returned rankings.

For removing the items corresponds to terms of lower informative involved in candidate check and similarity computation, we give an approximate form of ILSA similarity equation, defined as:

$${\text{sim}}_{\theta }\left(Q,d_{i}\right)=\frac{\sum_{t_{j}}\hat{Q}\left(t_{j}\right){\text{PartialSim}}_{\theta }\left(d_{i},t_{j}\right)}{\sqrt{\sum_{t_{j}}{\left(\hat{Q}\left(t_{j}\right)\right)}^{2}}}$$ (8)

where PartialSim_*θ*_(*d*_*i*_, *t*_*j*_) is the partial similarity under threshold *θ* between document *d*_*i*_ and term *t*_*j*_, defined as:

$${\text{PartialSim}}_{\theta }\left(d_{i},t_{j}\right)=\frac{V_{r}^{T}\left(t_{j},d_{i}\right)}{\sqrt{\sum_{t_{j}}{\left(V_{r}^{T}\left(t_{j},d_{i}\right)\right)}^{2}}}$$ (9)

if right-hand ≥ *θ*, PartialSim_*θ*_(*d*_*i*_, *t*_*j*_) = 0 for otherwise.

Under the threshold *θ*, we next consider removing the items corresponds to terms of lower informative from the partial index. Specifically, for a 2-tuple 〈*d*_*i*_,PartialSim(*d*_*i*_, *t*_*j*_)〉 in the corresponding partial index, we remove it from the partial index if the partial similarity PartialSim_*θ*_(*d*_*i*_, *t*_*j*_) is lower than *θ*. The partial index under threshold *θ* is denoted by a set $I_{\theta }=\cup_{i=1}^{\left|T\right|}\left\{I_{\theta }\left(t_{j}\right)\right\}$, where *I*_*θ*_(*t*_*j*_) = {〈*d*_*i*_,PartialSim_*θ*_(*d*_*i*_, *t*_*j*_)〉|*d*_*i*_ ∈ *D* ∧ PartialSim_*θ*_(*d*_*i*_, *t*_*j*_) ≠ *θ*}, i.e., only the 2-tuples of non-zero partial similarities are contained in *I*_*θ*_(*t*_*j*_). In which, 〈*d*_*i*_,PartialSim_*θ*_(*d*_*i*_, *t*_*j*_)〉 is a node of the partial index under threshold *θ* corresponds to the 2-tuple of 〈DocID,PartialSim〉 form, specifically, *d*_*i*_ is a document corresponds to DocID and PartialSim_*θ*_(*d*_*i*_, *t*_*j*_) is the partial similarity under threshold *θ* between document *d*_*i*_ and term *t*_*j*_ corresponds to PartialSim.

**Fig 2:**
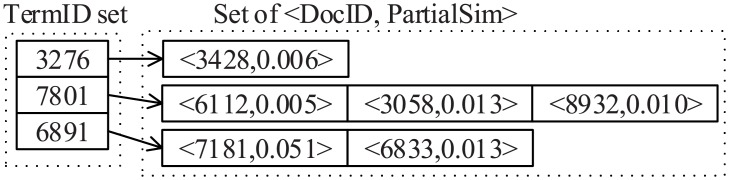
Example of partial index under *θ* = 0.005.

Fig 2 shows a partial index obtained from Fig 1 by setting threshold *θ* = 0.005. From this figure, we find that the index size is reduced after removing the 2-tuples 〈7181, 0.003〉, 〈1003, 0.001〉 and 〈9091, 0.001〉 correspond to the partial similarities lower than 0.005.

### Index building algorithm

The procedure for building partial index is shown in Algorithm 1. The input of this algorithm is matrix *V*_*r*_, document *D* and threshold *θ*, and the output is the partial index *I*_*θ*_. In the initialization step, the partial index *I*_*θ*_ is set as ∅. For each term $t_{j}\in \{t_{j}\left|\right|V_{r}^{T}\left(t_{j}:\right)\left|\neq 0\right\}$, we create node *I*_*θ*_(*t*_*j*_) initialized as ∅ in the partial index *I*_*θ*_. And for each document *d*_*i*_ ∈ *D*, we compute PartialSim_*θ*_(*d*_*i*_, *t*_*j*_) and create node 〈*d*_*i*_,PartialSim_*θ*_(*d*_*i*_, *t*_*j*_)〉 in *I*_*θ*_(*d*_*i*_) if PartialSim_*θ*_(*d*_*i*_, *t*_*j*_) ≥ *θ*.

**Algorithm 1** Algorithm for building partial index.

**Input:**

 Matrix *V*_*r*_, document set *D*, threshold *θ*;

**Output:**

 Partial index *I*_*θ*_;

1: Initialize *I*_*θ*_ as ∅;

2: **for** $t_{j}\in \{t_{j}\left|\right|V_{r}^{T}\left(t_{j}:\right)\left|\neq 0\right\}$ **do**

3:  *I*_*θ*_(*t*_*j*_)←∅;

4:  *I*_*θ*_ ← *I*_*θ*_ ∪ {*I*_*θ*_(*t*_*j*_)};

5:  **for** *d*_*i*_ ∈ *D* **do**

6: ${\text{PartialSim}}_{\theta }\left(d_{i},t_{j}\right)\leftarrow \frac{V_{r}^{T}\left(j,i\right)}{\sqrt{\sum_{j}{\left(V_{r}^{T}\left(j,i\right)\right)}^{2}}}$;

7:   **if** PartialSim_*θ*_(*d*_*i*_, *t*_*j*_) ≥ *θ* **then**

8:    *I*_*θ*_(*t*_*j*_)←*I*_*θ*_(*t*_*j*_) ∪ {〈*d*_*i*_,PartialSim_*θ*_(*d*_*i*_, *t*_*j*_)〉};

9:   **end if**

10:  **end for**

11: **end for**

12: **return** *I*_*θ*_;

Next we analyze the time complexity of this algorithm. In the initialization stage, the time cost for creating an empty set *I*_*θ*_ is derived as *O*(1). For each term *t*_*j*_, the time cost for computing partial similarities between *d*_*i*_ and *t*_*j*_ for all *d*_*i*_ ∈ *D* is derived as *O*(*N*). Since only the partial similarities bigger than *θ* are considering for creating index nodes, so the total time cost for creating 〈*d*_*i*_,PartialSim_*θ*_(*d*_*i*_, *t*_*j*_)〉 in *I*_*θ*_(*t*_*j*_) for all *d*_*i*_ ∈ *D* is derived as $O\left(\varepsilon_{t_{j}}N\right)$, where $\varepsilon_{t_{j}}$ is ratio of the partial similarities lower than *θ* between term *t*_*j*_ and all document *d*_*i*_ ∈ *D*. And then the total time cost for computing partial similarities and creating index nodes is derived as *O*((1 + *ϵ*)*N*). And finally, the time cost of this algorithm is derived as *O*(1 + (1 + *ϵ*)*rN*), where *ϵ* is average $\varepsilon_{t_{i}}$ for all $t_{j}\in \{t_{j}\left|\right|V_{r}^{T}\left(t_{j}:\right)\left|\neq 0\right\}$. The time cost of this algorithm is determined by the size of matrix $V_{r}^{T}$ and threshold *θ*. Usually, a higher threshold *θ* would reduce the searching space of ILSA, and subsequently lead to lower time cost of on-line query processing, since the partial similarities corresponds to lower partial similarities are skipped when building partial index.

### Index-based LSA (ILSA)

The on-line query processing procedure of the Index-based LSA (ILSA) is shown in Algorithm 2. For a given query *Q*, we transform it into a pseudo document vector $\hat{Q}$ and initialize 〈C,S〉 by setting both C and S as ∅, where C is the set candidate documents, S is the set of similarities between query and candidates, and the element $S(d_{i})$ in S is the similarity between query *Q* and document *d*_*i*_. And then, we search the candidate documents and compute the similarities between query and candidate documents by accumulating the partial similarities obtained from the partial index nodes corresponds to non-zero entries in $\hat{Q}$. Specifically, for each term $t_{j}\in \left\{t_{j}|\hat{Q}\left(t_{j}\right)\neq 0\right\}$, we get each document 〈*d*_*i*_,PartialSim_*θ*_(*d*_*i*_, *t*_*j*_)〉 ∈ *I*_*θ*_(*t*_*j*_), obtain partial similarity PartialSim_*θ*_(*d*_*i*_, *t*_*j*_) from 〈*d*_*i*_,PartialSim_*θ*_(*d*_*i*_, *t*_*j*_)〉, and then update the similarity between *Q* and *d*_*i*_ by accumulating $\frac{\hat{Q}\left(j\right){\text{PartialSim}}_{\theta }\left(d_{i},t_{j}\right)}{\sqrt{\sum_{j}{\left(\hat{Q}\left(j\right)\right)}^{2}}}$. GetSortedCenter(*k*, *Q*) is the function used for obtaining the *k* most similar documents according to 〈C,S〉, the basic process of which is that firstly get the *k* most similar nodes from C according to their corresponding similarities in S, then sort and return them.

**Algorithm 2** ILSA algorithm.

**Input**:

 Matrix *U*, $X=S_{r}^{-1}U_{r}^{T}$, index *I*_*θ*_, query *Q* and parameter *k*;

**Output**:

 Top-*r* most similar sorted documents;

1: Initialize 〈C,S〉 by setting C and S as ∅;

2: $\hat{Q}\leftarrow XQ$;

3: **For** $t_{j}\in \left\{t_{j}|\hat{Q}\left(t_{j}\right)\neq 0\right\}$ **do**

4:  **For** 〈*d*_*i*_,PartialSim_*θ*_(*d*_*i*_, *t*_*j*_)〉 ∈ *I*_*θ*_(*t*_*j*_) **do**

5:   obtain PartialSim_*θ*_(*d*_*i*_, *t*_*j*_) from 〈*d*_*i*_,PartialSim_*θ*_(*d*_*i*_, *t*_*j*_)〉;

6:   **if** $d_{i}\in C$ **then**

7:    $S\left(d_{i}\right)\leftarrow S\left(d_{i}\right)+\frac{\hat{Q}\left(j\right){\text{PartialSim}}_{\theta }\left(d_{i},t_{j}\right)}{\sqrt{\sum_{j}{\left(\hat{Q}\left(j\right)\right)}^{2}}}$;

8:   **else**

9:    $S\left(d_{i}\right)\leftarrow \frac{\hat{Q}\left(j\right){\text{PartialSim}}_{\theta }\left(d_{i},t_{j}\right)}{\sqrt{\sum_{j}{\left(\hat{Q}\left(j\right)\right)}^{2}}}$;

10:    $C\leftarrow C\cup \{d_{i}\}$;

11:    $S\leftarrow S\cup \{S\left(d_{i}\right)\}$;

12:   **end if**

13:  **end for**

14: **end for**

15: **return** GetSortedCenter(k,〈C,S〉);

The time cost of this algorithm is affected by the following three aspects. First is the time cost for transforming the given query into pseudo document, derived as *O*(*rN*). Second is the time cost for choosing top *k* most similar documents, derived as $O(\sum_{t_{j}\in \left\{t_{j}|\hat{Q}\left(t_{j}\right)\neq 0\right\}}|I_{\theta }\left(t_{j}\right)|+k|C|)$. And third is the time cost for sorting these *k* documents, denoted by *O*(Γ(*k*)), that is depends on the sort algorithm and we use selection sort in our research. So the total time cost of this algorithm is derived as $O(rN+\sum_{t_{j}\in \left\{t_{j}|\hat{Q}\left(t_{j}\right)\neq 0\right\}}|I_{\theta }\left(t_{j}\right)|+k|C|+\Gamma \left(k\right))$.

In ILSA algorithm, we first get the non-zero entries from vector $\hat{Q}$, and then check the candidates in the partial index corresponds to the non-zero entries. Therefore, the candidate set is derived as $C=\cup_{t_{j}\in \left\{t_{j}|\hat{Q}\left(t_{j}\right)\neq 0\right\}}C\left(t_{j}\right)$, where $C(t_{j})$ is the sub candidate set corresponds to term *t*_*j*_. We access only the 2-tuple 〈*d*_*i*_,PartialSim_*θ*_(*d*_*i*_, *t*_*j*_)〉 ∈ *I*_*θ*_(*t*_*j*_) in partial index *I*_*θ*_ during on-line query processing, so the sub candidate set $C(t_{j})$ is derived as $C\left(t_{j}\right)=\left\{d_{i}|\langle d_{i},{\text{PartialSim}}_{\theta }\left(d_{i},t_{j}\right)\rangle \in I_{\theta }\left(t_{j}\right)\right\}$, and subsequently the candidate set C is derived as $C=\cup_{t_{j}\in \left\{t_{j}|\hat{Q}\left(t_{j}\right)\neq 0\right\}}\left\{d_{i}|\langle d_{i},{\text{PartialSim}}_{\theta }\left(d_{i},t_{j}\right)\rangle \in I_{\theta }\left(t_{j}\right)\right\}$. When giving a higher threshold *θ*, the accumulation operations for computing similarities would be reduced, which consequently reduces the time cost. In this case, the size of $C(t_{j})$ would become smaller, and hence the size of C would have a downward trend. So the time cost for choosing the *r* centers from C would become lower as well. Note that the size of $C(t_{j})$ is equal to the size of *I*_*θ*_(*t*_*j*_).

**Lemma 1** *For given document* *d*_*i*_ ∈ *D*, *term* *t*_*i*_ ∈ *T* *and threshold* *θ*, *we have* 0 ≤ PartialSim(*d*_*i*_, *t*_*j*_) − PartialSim_*θ*_(*d*_*i*_, *t*_*j*_) ≤ *θ*.

*Proof*. By Eqs (7) and (9), we have PartialSim(*d*_*i*_, *t*_*j*_) = PartialSim_*θ*_(*d*_*i*_, *t*_*j*_) when PartialSim(*d*_*i*_, *t*_*j*_) > *θ*, which gives PartialSim(*d*_*i*_, *t*_*j*_) − PartialSim_*θ*_(*d*_*i*_, *t*_*j*_) = 0; and when PartialSim(*d*_*i*_, *t*_*j*_) ≤ *θ*, we have PartialSim_*θ*_(*d*_*i*_, *t*_*j*_) = 0, which gives PartialSim(*d*_*i*_, *t*_*j*_) − PartialSim_*θ*_(*d*_*i*_, *t*_*j*_) = PartialSim(*d*_*i*_, *t*_*j*_) ≤ *θ*.

**Theorem 1** *For given query* *Q*, *document* *d*_*i*_ ∈ *D* *and threshold* *θ*, *we have* 0 ≤ sim(*Q*, *d*_*i*_) − sim_*θ*_(*Q*, *d*_*i*_) ≤ *θ*.

*Proof*. For given query *Q*, document *d*_*i*_ ∈ *D* and threshold *θ*, by Eqs (6) and (8), we have

$$\begin{array}{cc} \text{sim}\left(Q,d_{i}\right)-{\text{sim}}_{\theta }\left(Q,d_{i}\right) & =\frac{\sum_{t_{j}}\hat{Q}\left(t_{j}\right)\text{PartialSim}\left(d_{i},t_{j}\right)}{\sqrt{\sum_{t_{j}}{\left(\hat{Q}\left(t_{j}\right)\right)}^{2}}}-\frac{\sum_{t_{j}}\hat{Q}\left(t_{j}\right){\text{PartialSim}}_{\theta }\left(d_{i},t_{j}\right)}{\sqrt{\sum_{j}{\left(\hat{Q}\left(j\right)\right)}^{2}}}\\ & =\frac{\sum_{t_{j}}\hat{Q}\left(t_{j}\right)}{\sqrt{\sum_{t_{j}}{\left(\hat{Q}\left(j\right)\right)}^{2}}}\left(\text{PartialSim}\left(d_{i},t_{j}\right)-{\text{PartialSim}}_{\theta }\left(d_{i},t_{j}\right)\right) \end{array}$$

By Lemma 1, we have 0 ≤ PartialSim(*d*_*i*_, *t*_*j*_) − PartialSim_*θ*_(*d*_*i*_, *t*_*j*_) ≤ *θ*, so sim(*Q*, *d*_*i*_) − sim_*θ*_(*Q*, *d*_*i*_) ≥ 0 and

$$\begin{array}{cc} \text{sim}\left(Q,d_{i}\right)-{\text{sim}}_{\theta }\left(Q,d_{i}\right) & \leq \frac{\sum_{t_{j}}\hat{Q}\left(t_{j}\right)}{\sqrt{\sum_{t_{j}}{\left(\hat{Q}\left(t_{j}\right)\right)}^{2}}}\cdot \theta =\frac{\sum_{t_{j}}\hat{Q}\left(t_{j}\right)\cdot \theta }{\sqrt{\sum_{t_{j}}{\left(\hat{Q}\left(t_{j}\right)\right)}^{2}}\sqrt{\theta^{2}}}\cdot \theta \leq \theta \end{array}$$

Theorem 1 gives the maximal difference of the maximal upper bound between LSA and ILSA, which is under control by tuning threshold *θ*.

## Results

In this section, some preliminary experimental results are reported in real datasets. Experiments were done on a 2.90 GHz Intel(R) Core i7-3520M CPU with 8 GB main memory, running Windows 7 SP1. All algorithms were implemented in C++ and compiled by using Visual Studio C++. Net 2010.

### Datasets and evaluation

The dataset used in our experiments is the set of the papers that are selected from DBLP (http://dblp.uni-trier.de/). We only keep entries of the snapshot that correspond to the papers published before March 10th, 2013. The titles of the papers that are published in SIGMOD, VLDB, SIGIR, CIKM, ICDE and EDBT conferences from 2004 to 2013 are selected. From this dataset, we choose the titles of 8,884 papers to test our algorithm and the comparisons, which contains 8,572 terms after removing the stop words, and the values of entries in term-document matrix is assigned by the TF*IDF model [53, 54].

We use the NDCG (Normalized Discounted Cumulative Gain) [59] to evaluate the effectiveness of returned ranking list. The NDCG@*k* (NDCG value at the *k*-th position) of the ranking result is computed by the exact LSA scores. Formally, NDCG@*k* is defined as:

$$\text{NDCG}@k=\frac{\text{DCG}@k}{\text{IDCG}@k}$$ (10)

where DCG@k (Discounted Cumulative Gain at *k*) is defined as:

$$\text{DCG}@k=\left\{\begin{array}{cc} & \text{REL}(v,v_{i}),\text{if}\;i<2\\ & \text{DCG}@i+\sum_{i=2}^{k}\frac{\text{REL}(v,v_{i})}{\log_{2}i},\text{if}\;i\geq 2 \end{array}\right.$$ (11)

where *i* denotes position of *v*_*i*_ in the returned list, REL(*v*, *v*_*i*_) denotes the similarity score of the naive LSA between *v* and *v*_*i*_.

Efficiency comparison includes the running time for building index, execution time of on-line query processing. In [60], extensive experiments are done in large datasets to test the performance of LSA. The results suggest that, a value *r* ≈ 400 provides the best performance, and there is something of an “island of stability” in the *r* = 300 to 500 range. According to this conclusion, we set parameter *r* = 400 to test both LSA and ILSA in our experiments. Other parameter settings of the comparison method are implemented strictly following the literature. We input 10 queries that consists of two keywords to test the NDCG value and the time cost of on-line query processing. In order to accurately test the execution time of query processing, we process each query with 10 runs, and then average the total time cost.

### Effectiveness

In this section, we observe effectiveness of ILSA through testing the NDCG value by setting different threshold *θ*, and then choose different *k* and *r* to observe the NDCG value on a fixed *θ*.

**Fig 3:**
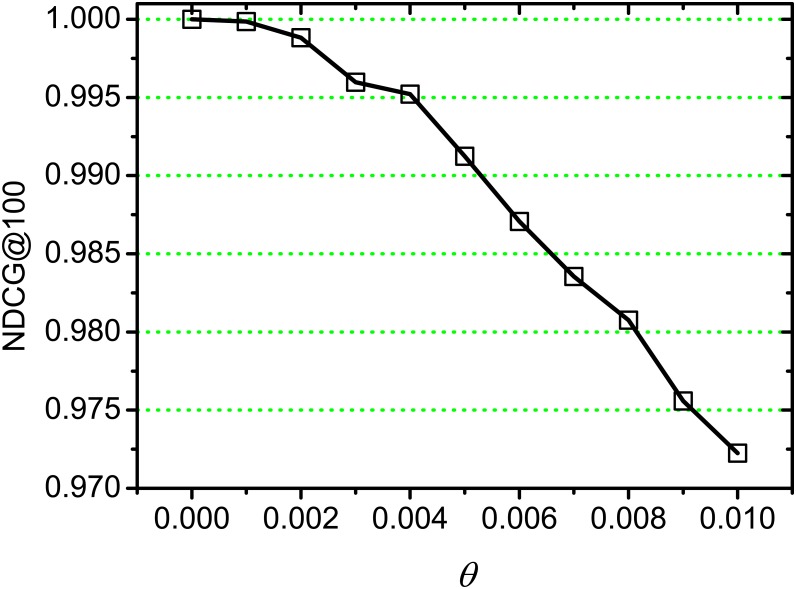
NDCG on varying *θ*.

Fig 3 shows NDCG values on varying threshold *θ* and the interval is 0.001, where *k* is set as 100. From *θ* = 0 to 0.010, we observe that NDCG value decreases with *θ* increasing, this is because higher *θ* would lead to more accuracy loss, which is consistent with our previous discussions in Theorem 1. We also observe that the accuracy loss of ILSA before *θ* = 0.01 is not too much, which suggests a good ranking quality of our approach.

**Fig 4:**
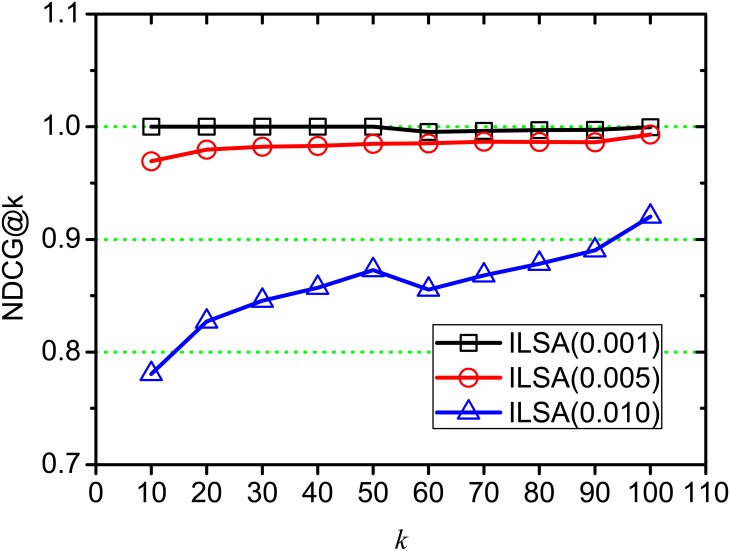
NDCG on varying *k*.

Fig 4 shows the NDCG change on different position *k*, where *θ* = 0.001, 0.005, 0.010, and ILSA(0.001), ILSA(0.005), ILSA(0.010) represent the ILSA algorithms at *θ* = 0.001, 0.005, 0.010 respectively. With *k* increasing, we find that the curve of ILSA(0.001) is nearly horizontal, since the accuracy loss is very minor; and the NDCG values of ILSA(0.005), ILSA(0.010) shown a upward generally as *k* increases, this is because some similar documents are lost when setting a higher threshold *θ*. And these similar documents are obtained again as *k* increases, which increases the accuracy loss. At each position *k*, the NDCG value of ILSA(0.001) is always close to 1, and ILSA(0.005) is lower than ILSA(0.001) and higher than ILSA(0.010), since higher *θ* leads to more accuracy loss, which is consistent with the result in Fig 3. The NDCG of both LSA and ILSA(0) are always 1 at each position *k*, which are not repeatedly shown in our experiment.

**Fig 5:**
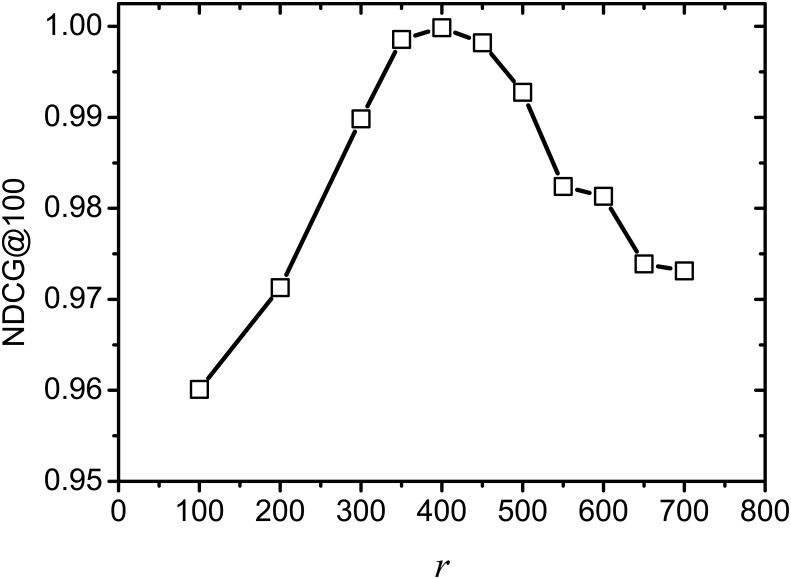
NDCG on varying *r*.

Fig 5 shows the NDCG change of ILSA on varying rank *r*, where *k* = 100 and *θ* = 0.001. From this result, we observe that the NDCG increases rapidly from *r* = 100 to 350, this is because more informative terms are contained in similarity computation when increasing *r*, which consequently increases the effectiveness of the returned rankings. From *r* = 350 to 450, the NDCG scores are relatively higher and stable, since the number of informative terms are suitable and the noisy terms are not too many. After *r* = 450, the NDCG value shows a downward trend, since the number of noisy terms are increased when *r* is set too big, which also affects the returned rankings. This result demonstrates that the returned rankings of ILSA are affected evidently by *r*, and the effectiveness would be decreased when *r* is set too big or too small.

### Efficiency

**Fig 6:**
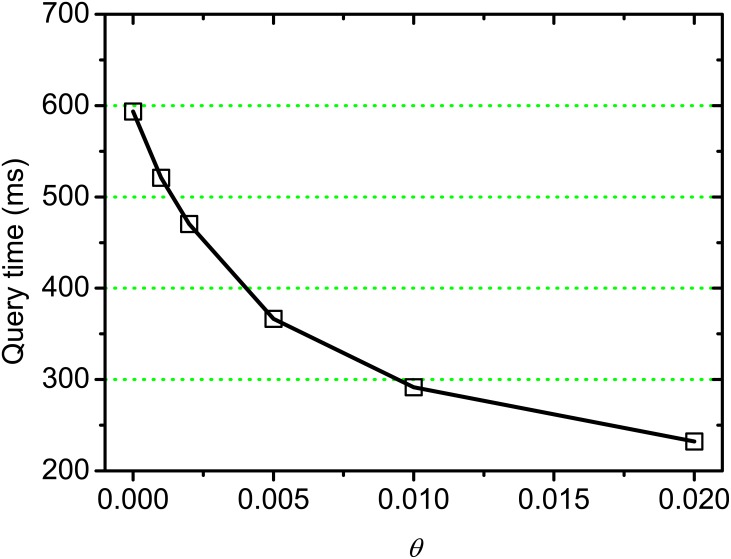
Query processing time on varying *θ*.

Fig 6 shows the execution time of on-line query processing on varying *θ*, where *k* = 100. From this result, we observe that the time cost decreases with *θ* increasing, this is because the index nodes corresponds to the partial similarities lower than threshold *θ* are skipped when building partial index, and subsequently the searching space of on-line query processing is reduced.

**Fig 7:**
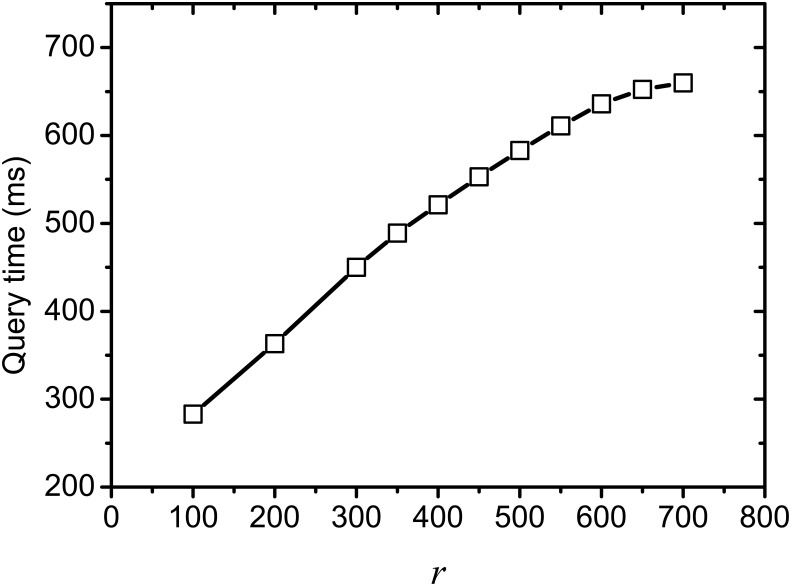
Query processing time on varying *r*.

Fig 7 shows the time cost of on-line query processing on varying rank *r*, where *k* = 100 and *θ* = 0.001. We observe that the execution time of on-line query processing increases with *r* increasing, this is because more operations on transformation from the query into pseudo document are involved during on-line query processing. And the incremental time becomes smaller as gradually as *r* increases, since the size of the document set corresponds to each term in partial index is reduced, which reduces the operations for checking candidates during on-line query processing.

**Fig 8:**
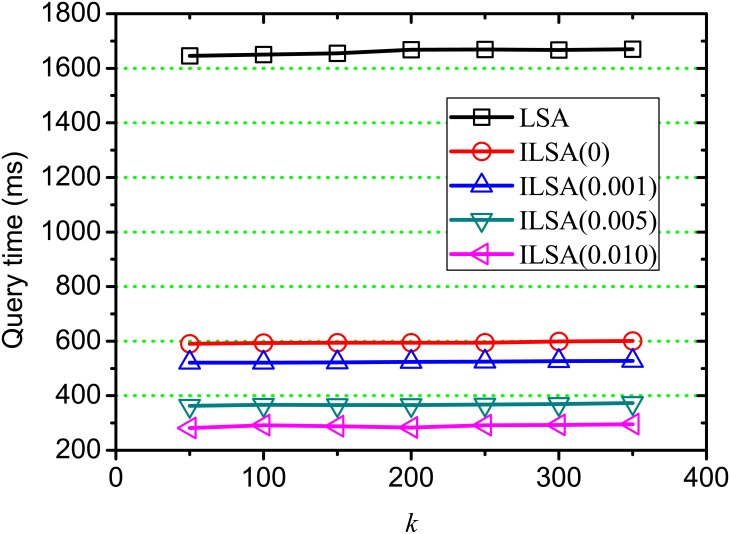
Query processing time on varying *k*.

Fig 8 shows the execution time of on-line query processing on varying *k*, where *θ* = 0, 0.001, 0.005, 0.010 and *k* = 50, 100, 150, …, 450. From this result, we observe that the incremental time is very minor as *k* increases, this is because the time cost of on-line query processing is mainly affected by the similarity computation between query and candidates and the transformation from the query into the pseudo document vector. Both of these two steps account for a large proportion of the time cost during on-line query processing. ILSA(0.010) is the most efficient method, this is because the searching space is reduced during on-line query processing when setting a higher *θ*, which is consistent with the result in Fig 6. Generally, our proposed ILSA is more efficient than LSA at different position *k*, which demonstrates the improvement on efficiency of our proposed ILSA.

**Fig 9:**
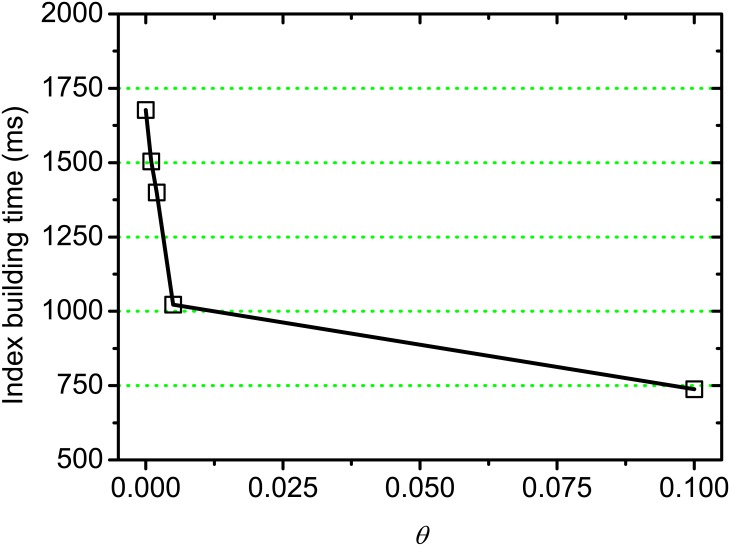
Index building time on varying *θ*.

Fig 9 shows the time cost for building partial index on varying threshold *θ*. We observe that the time cost for building partial index decreases with threshold *θ* increasing, this is because the operations for creating index nodes are saved by skipping the partial similarities lower than *θ*, which is consistent with the previous discussions on complexity analysis. This result demonstrates that the additional time cost in preprocessing stage for building partial index is very low, which would benefit some researches on semantic analysis in real applications.

**Fig 10:**
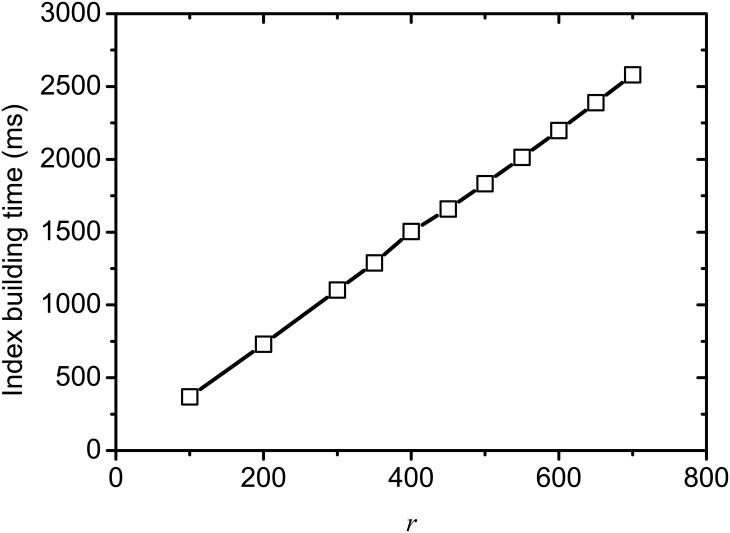
Index building time on varying *r*.

Fig 10 shows the time cost for building partial index on varying rank *r*, where *k* = 100 and *θ* = 0.001. From this figure, we find that the time cost of index building increases linearly with *r* increasing, since a bigger *r* can increase the size of SVD matrices and consequently increase access operations to these matrices, which is consistent with the analysis on time complexity in Algorithm 1.

### Scalability

**Fig 11:**
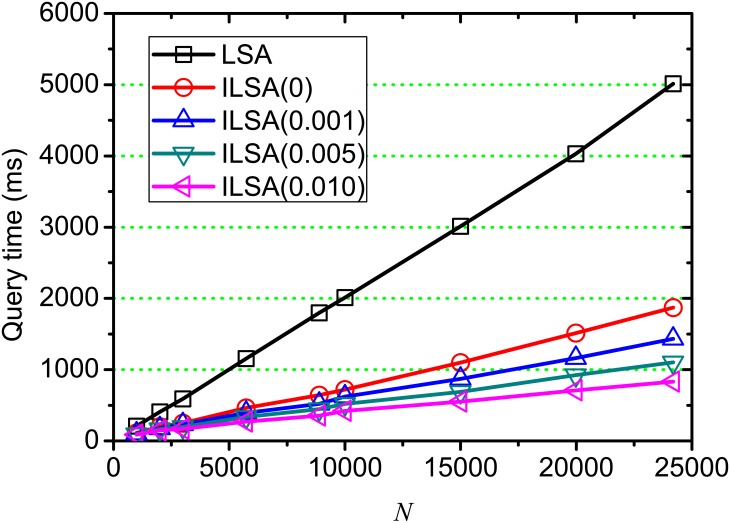
On-line query processing time on varying *N*.

Fig 11 shows the execution time of on-line query processing on different document scale *N*, where *θ* = 0, 0.001, 0.005, 0.010 respectively. We observe that the query processing time increases when document scale grows large, since the incremental documents increase the searching space over partial index during on-line query processing. We also observe that the execution time of ILSA(0) is higher than others, and ILSA(0.010) is the most efficient one, since the operations for computing similarities and checking candidates are reduced by setting a higher threshold *θ*.

**Fig 12:**
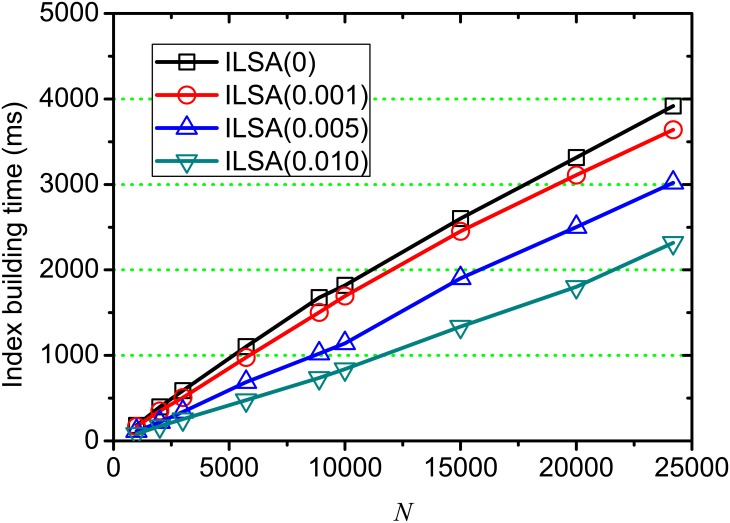
Index building time on varying *N*.

Fig 12 shows the index building time on different document scale *N*, where *θ* = 0, 0.001, 0.005, 0.010 respectively. From this figure, we observe that the time cost for building index increases linearly with *N* increasing, since more access operations on matrices of SVD are involved in the index building process. In practice, although the running time is significantly higher than the query processing time at each document scale, it is acceptable in real applications since the index is built in off-line stage.

**Fig 13:**
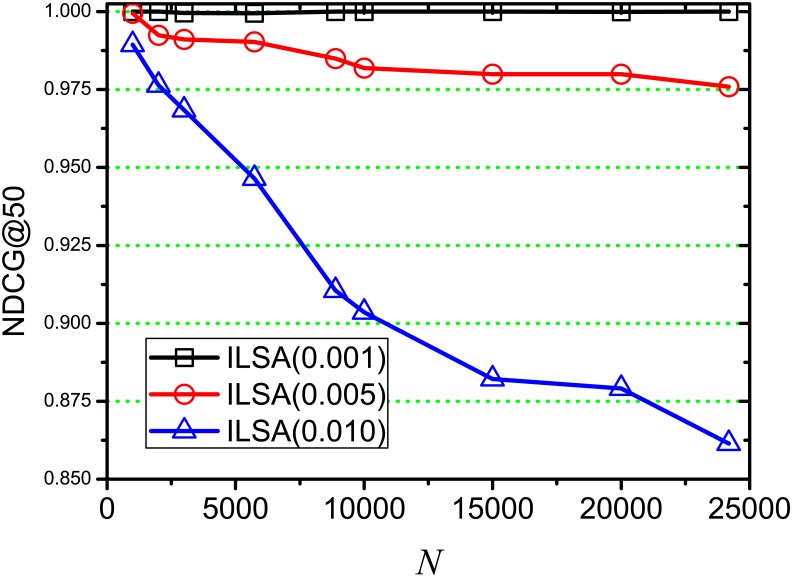
NDCG on varying *N*.

Fig 13 shows the NDCG change on different document scale *N*, where *θ* = 0.001, 0.005, 0.010. We find that the NDCG value of ILSA(0.001) is always close to 1 on varying *N* and the change is minor, which shows good performance when searching similar documents. The NDCG value of ILSA(0.005) shows a minor downward trend with *N* increasing, since the candidate set increases when increasing document scale, which subsequently increases the number of similar documents to the given query, but the similar documents should be returned are lost when setting a bigger *θ*. The downward trend of ILSA(0.010) is more evident than both ILSA(0.001) and ILSA(0.005) as *N* increases, since *θ* = 0.010 leads to more accuracy loss when compared to *θ* = 0.001, 0.005. We also find that the curve of ILSA(0.010) of is evidently lower than both ILSA(0.001) and ILSA(0.005), this is because the effectiveness of the returned rankings would be decreased when setting a higher threshold *θ*, which is consistent with the result in Fig 3.

## Discussion

This paper introduced an index-based query processing algorithm ILSA for efficiently finding similar documents in large document datasets. Compared to the LSA algorithm, ILSA searches the documents over a designed partial index that is derived from the SVD of the term-document matrix, and the searching space can be reduced by skipping the partial similarities lower than a given threshold. ILSA reduces the time cost of on-line query processing by pruning the candidate documents that are not promising and skipping the operations that make little contribution to similarity scores, which shows better performance than LSA, and the accuracy loss is under controlled by tuning the threshold. Empirical studies on DBLP through comparison with LSA demonstrate the effectiveness and efficiency of our approach.

There are some directions in our future work. First, ILSA is on the static datasets, and the dynamic datasets are not considered. Accordingly, we will study on how to building a dynamical partial index for the dynamic term set and document set by integrating existing incremental LSA algorithm [61, 62] and incremental SVD algorithm [63–65]. Second, our approach does not pay attention to the transformation process from query into pseudo document which involves lots of unnecessary operations on entries of lower values and increases the execution time of on-line query process. To further reduce the time cost of on-line query process, we plan to optimize the transformation process by skipping the entries of lower values in the SVD matrices, and further optimize the similarity computation between query and candidate documents by skipping the entries of lower values in the vector of pseudo document.
